# Supplementary material for: From sequence to enzyme mechanism using multi-label machine learning
Source: BMC Bioinformatics. 2014 May 19;15:150. doi: 10.1186/1471-2105-15-150 (PMC4229970; doi:10.1186/1471-2105-15-150)
Supplement: Additional file 2 — Java code of ml2db. Additional file ml2db_code.tar.gz contains the Java source code to run the multi-label machine learning experiments and save the results to database. The code’s Javadoc is included. [file 1471-2105-15-150-S2.zip › additional file 2/ml2db/ml2db/doc/mulan/evaluation/measure/package-use.html]

Uses of Package mulan.evaluation.measure


JavaScript is disabled on your browser.


- Overview
- Package
- Class
- Use
- Tree
- Deprecated
- Index
- Help

- Prev
- Next

- Frames
- No Frames

- All Classes

# Uses of Package mulan.evaluation.measure

- Packages that use mulan.evaluation.measure

  | Package | Description |
  | --- | --- |
  |  |  |
  | --- | --- |
  | mulan.classifier.meta.thresholding |  |
  | mulan.data |  |
  | mulan.evaluation |  |
  | mulan.evaluation.measure |  |
- Classes in mulan.evaluation.measure used by mulan.classifier.meta.thresholding

  Class and Description

  BipartitionMeasureBase

  ExampleBasedBipartitionMeasureBase

  Base class for example-based bipartition measures
- Classes in mulan.evaluation.measure used by mulan.data

  Class and Description

  Measure

  Interface for a measure, used to evaluate the performance of a multi-label learner
  on when performing multi-label learning task.
- Classes in mulan.evaluation.measure used by mulan.evaluation

  Class and Description

  Measure

  Interface for a measure, used to evaluate the performance of a multi-label learner
  on when performing multi-label learning task.
- Classes in mulan.evaluation.measure used by mulan.evaluation.measure

  Class and Description

  BipartitionMeasureBase

  ConfidenceMeasureBase

  ExampleBasedBipartitionMeasureBase

  Base class for example-based bipartition measures

  LabelBasedAUC

  Implementation of the label-based macro precision measure.

  LabelBasedAveragePrecision

  LabelBasedBipartitionMeasureBase

  Base class for label-based bipartition measures

  LabelBasedFMeasure

  Base implementation of the label-based macro/micro f-measures.

  LabelBasedPrecision

  Base implementation of the label-based macro/micro precision measures.

  LabelBasedRecall

  Common class for the micro/macro label-based recall measures.

  LabelBasedSpecificity

  Common class for the micro/macro label-based recall measures.

  LossBasedBipartitionMeasureBase

  LossBasedRankingMeasureBase

  MacroAverageMeasure

  Interface for macro average measures

  MeanAverageInterpolatedPrecision

  Implementation of MAiP (Mean Average Interpolated Precision)

  MeanAveragePrecision

  Implementation of MAP (Mean Average Precision)

  Measure

  Interface for a measure, used to evaluate the performance of a multi-label learner
  on when performing multi-label learning task.

  MeasureBase

  RankingMeasureBase

- Overview
- Package
- Class
- Use
- Tree
- Deprecated
- Index
- Help

- Prev
- Next

- Frames
- No Frames

- All Classes
